# Zebrafish as model organism for cNMP research

**DOI:** 10.1186/2050-6511-16-S1-A45

**Published:** 2015-09-02

**Authors:** Fanni Dittmar, Salim Seyfried, Volkhard Kaever, Roland Seifert

**Affiliations:** 1 Institute of Pharmacology, Hannover Medical School (MHH), Hannover, Germany; 2 Institute of Biochemistry and Biology, University of Potsdam, Potsdam, Germany; 3 Research Core Unit Metabolomics, Institute of Pharmacology, Hannover Medical School (MHH), Hannover, Germany

## Introduction

The zebrafish *Danio rerio* has become an important vertebrate model organism for a wide range of scientific research questions including development, genetics, and disease [1]. The zebrafish is particularly well suited for this research due to its small size, rapid development, short generation time, optical transparency of embryos and larvae as well as functional conservation of genes [1]. Furthermore, application of drugs is easy since zebrafish readily absorb compounds from their surrounding media [2].

The aim of our study was to determine the composition and to quantify the endogenous level of different cyclic nucleotides (cNMPs) in various developmental stages and organs of *Danio rerio*.

## Methods

Wild-type AB zebrafish were mated; for tissue preparation, we harvested embryos at 24 hours post fertilization (hpf) and larvae at 5 days post fertilization (dpf). Organs (eyes, brain, heart, entrails, eggs and testes) from adult female and male wild-type AB zebrafish were dissected according to the guidance of Gupta and Mullins [3]. All samples were then prepared for measurement of cNMP concentrations via a highly sensitive and specific method, namely high-performance liquid chromatography tandem mass spectrometry (HPLC-MS/MS) [4].

## Results

We could detect all studied cNMPs (namely cAMP, cCMP, cGMP, cIMP and cUMP) with specific variations as outlined below. (1) cGMP and cAMP could be detected in tissue samples of both developmental stages and within all harvested organs. Remarkably, they are the only cyclic nucleotides detected in brain and entrails. The highest cGMP concentrations were present in eyes; the highest cAMP concentrations in larvae tissue. (2) cIMP was only detected in some organs with the highest concentration in heart. (3) cCMP could not be detected in embryos but in larvae and within several organs. Like cIMP, the highest cCMP concentrations were present in heart. (4) cUMP was detected in both developmental stages as well as in some organs. This cNMP was most abundant within the heart.

## Conclusion

The zebrafish *Danio rerio* constitutes a good model organism not only for studies focused on development, genetics and disease but also in the field of cyclic nucleotides, especially related to the role of cNMPs in cardiovascular biology. We observed specific cNMP patterns in development and in different organs, which is in support of the hypothesis of a distinct cNMP signaling code [5].

**Table 1:** cNMP concentrations of zebrafish embryos, larvae and various organs.

n=2	cGMP[pmol/mg protein]	cIMP[pmol/mg protein]	cAMP[pmol/mg protein]	cCMP[pmol/mg protein]	cUMP[pmol/mg protein]
**Embryos 24 hpf**	0.282 ± 0.009	not detected	2.29 ± 0.37	not detected	0.414 ± 0.067
**Larvae 5 dpf**	1.99 ± 0.32	not detected	23.66 ± 3.97	0.328 ± 0.119	0.890 ± 0.422
**Eyes**	2.69 ± 0.03	0.111 ± 0.000	2.73 ± 0.34	0.043 ± 0.004	0.087 ± 0.040
**Brain**	0.149 ± 0.073	not detected	10.02 ± 2.89	not detected	not detected
**Heart**	0.607 ± 0.124	0.452 ± 0.069	23.12 ± 17.40	0.504 ± 0.143	1.11 ± 0.10
**Entrails**	0.292 ± 0.031	not detected	16.52 ± 2.30	not detected	not detected
**Eggs**	0.002 ± 0.003	not detected	0.29 ± 0.02	0.004 ± 0.001	0.008 ± 0.001
**Testes**	0.802 ± 1.134	0.407 ± 0.037	12.15 ± 3.80	0.116 ± 0.164	0.617 ± 0.052

## Future studies

Zebrafish embryos will be treated with the NO-independent sGC activator cinaciguat (BAY 58-2667) and the NO-synergistic sGC stimulator 3-(4-amino-5-cyclopropylpyrimidine-2-yl)-1-(2-fluorobenzyl)-1H-pyrazolo[3,4-b]pyridine (BAY 41-2272), from one day post fertilization to five days post fertilization. cNMP concentrations and morphological changes will be determined afterwards. Another research area will be the elucidation of the controversial role of cIMP as second messenger [6,7].

## References

[B1] Lawrence C The husbandry of zebrafish (Danio rerio): A review Aquaculture 2007 269 1-4 1 20 10.1016/j.aquaculture.2007.04.077

[B2] Langheinrich U Zebrafish: A new model on the pharmaceutical catwalk BioEssays 2003 25 9 904 912 10.1002/bies.10326 12938180

[B3] Gupta T Mullins MC Dissection of organs from the adult zebrafish J Vis Exp 2010 37 10.3791/1717 PMC3144575 20203557

[B4] Bähre H Danker KY Stasch JP Kaever V Seifert R Nucleotidyl cyclase activity of soluble guanylyl cyclase in intact cells Biochem Biophys Res Commun 2014 443 4 1195 1199 10.1016/j.bbrc.2013.12.108 24380860

[B5] Seifert R Schneider EH Bähre H From canonical to non-canonical cyclic nucleotides as second messengers: Pharmacological implications Pharmacol Ther 2015 148 154 184 25527911 10.1016/j.pharmthera.2014.12.002

[B6] Chen Z Zhang X Ying L Dou D Li Y Bai Y cIMP synthesized by sGC as a mediator of hypoxic contraction of coronary arteries AJP Hear Circ Physiol 2014 307 3 H328 H336 10.1152/ajpheart.00132.2014 24906916

[B7] Seifert R Is cIMP a second messenger with functions opposite to those of cGMP? Naunyn Schmiedebergs Arch Pharmacol 2014 387 9 897 899 10.1007/s00210-014-1013-1 25017018

